# Measuring Tidal Volume with Diaphragm Movement and Chest Circumference

**DOI:** 10.1298/ptr.E10318

**Published:** 2025-03-13

**Authors:** Akihiro KAKUDA

**Affiliations:** Department of Physical Therapy, Morinomiya University of Medical Sciences, Japan

**Keywords:** Diaphragm, Tidal volume, Ultrasound

## Abstract

Objective: The movement of the diaphragm plays an important role in respiration. In this study, we proposed and validated a new method for estimating the volume of a single ventilation (representing the tidal volume [VT]) from the vertical distance of diaphragmatic movement and thoracic circumference. Method: Diaphragm excursion (DE) was measured in healthy adult subjects via ultrasound, and the thoracic cavity volume change was estimated based on DE and thoracic circumference. Moreover, we measured the VT obtained by an expiratory gas analyzer and examined the relationships between DE and thoracic volume change (TVC) and between DE and VT. Results: The results showed that a correlation (ρ = 0.609) existed between DE and VT, and an even higher correlation existed between TVC and VT. TVC correlated better with the product of thoracic circumference squared and DE (ρ = 0.839) than with the product of thoracic circumference and DE as an alternative index (ρ = 0.746). Conclusion: Our findings demonstrate that, taking into account body size in addition to DE, changes in thoracic cavity volume are useful predictors of VT and provide an alternative measure for assessing the respiratory function, which will improve clinical and research practice in respiratory care.

## Introduction

Respiration is a fundamental function that is essential to support life, and its efficient management and assessment are important in clinical medicine. The diaphragm is a muscle that plays a central role in respiration, and its movement reflects respiratory volume^1)^. Respiratory function assessment is an important component of respiratory rehabilitation programs. One of the goals of a respiratory rehabilitation program is to improve tidal volume (VT), which is expected to improve the patient’s quality of life^2,3)^.

Respiratory characteristics are quantified in terms of the volume of ventilation, respiratory rate (RR), and inspiratory/expiratory time ratio (IE ratio). These parameters reflect lung function and are indicators of the body’s metabolic state and gas exchange efficiency^4)^.

However, the clinical significance of VT estimation extends beyond the basic lung function metrics. Passive assessments, like lung capacity and RR, are valuable, but VT estimation provides critical insights into a patient’s respiratory mechanics and ventilatory capacity. This is particularly relevant for patient populations during postoperative recovery, critical care, or those with chronic respiratory diseases, where continuous, noninvasive monitoring of respiratory function is essential.

Spirometers and other instruments are widely used for VT measurement; however, they require patients to actively perform specific breathing maneuvers, which can pose challenges^4)^. While some may argue that attaching a mask is relatively effortless, achieving consistent compliance and accuracy is not guaranteed, especially in older populations or those with neuromuscular disorders. Thus, there is a pressing need for simpler, more reliable methods that minimize patient effort and still yield accurate measurements.

In recent years, ultrasound-based techniques for observing diaphragmatic movement have been developed, offering a noninvasive and real-time approach to assessing respiratory function. These techniques include measuring diaphragm muscle thickness and diaphragm excursion (DE). These reflect respiratory muscle strength and thoracic volume changes (TVCs), respectively. DE, in particular, has shown promise as a predictor of VT. The evaluation of diaphragm muscle thickness is related to respiratory muscle strength^5)^, and its usefulness as an indicator for ventilator withdrawal in the Intensive Care Unit has been reported. This is because it can be used to evaluate respiratory function even when a patient is not awake^6,7)^. DE enables observation of the changes in thoracic cavity volume, by visualizing the dynamics of diaphragm contraction from the body surface, in real time. Previous studies that measured DE using ultrasound^8,9)^ reported that it could be a predictor of VT. Yamada et al.^10)^ also examined the relationship between DE and ventilation volume using dynamic X-ray. By contrast, they reported that individual physiological characteristics such as body size, respiratory muscle strength, and lung capacity may influence the measured values of DE and VT. Additionally, adipose tissue thickness and other factors are known to affect visualization of the diaphragm by ultrasound. These characteristics may vary in different individuals and cause variations in the accuracy of DE measurements. Therefore, when evaluating the relationship between DE and VT, it is necessary to consider not only the measured DE values but also other variables such as body size and physiological characteristics.

This study aimed to address these challenges by developing a method that estimates VT using DE and thoracic circumference, accounting for body size. The approach was based on a geometric model that approximated TVCs, potentially enhancing the accuracy of VT prediction while reducing patient effort. By simultaneously measuring DE with ultrasound and VT, using an expiratory gas analyzer in healthy individuals, this research sought to establish a reliable, non-effort-dependent method for clinical and research applications.

## Methods

### Sample size determination and participants

A power analysis was conducted to determine the appropriate sample size for this study. Based on preliminary experiments and previous studies^9)^, we assumed a moderate to strong correlation (ρ = 0.65), and performed sample size estimation with a significance level of 0.05. This analysis indicated that 15 participants would provide adequate statistical power for detecting significant correlations. Accordingly, 15 healthy young adults (9 women and 6 men) were recruited for this study. Participants had a mean age of 20.7 ± 1.0 years, height of 164.6 ± 8.2 cm, weight of 55.4 ± 5.4 kg, and body mass index of 20.4 ± 1.6 kg/m2. Individuals with a history of smoking or respiratory disease were excluded. All participants provided written informed consent after a detailed explanation of the study’s purpose and procedures. This study was approved by the Ethics Committee of Morinomiya University of Medical Sciences (Approval No. 2019-052).

### Measurement procedures

VT was measured using an expiratory gas analyzer (AE100i; Minato Medical Science, Osaka, Japan) with the patient in the seated position, using the breath-by-breath method during resting breathing.

**Fig. 1.**
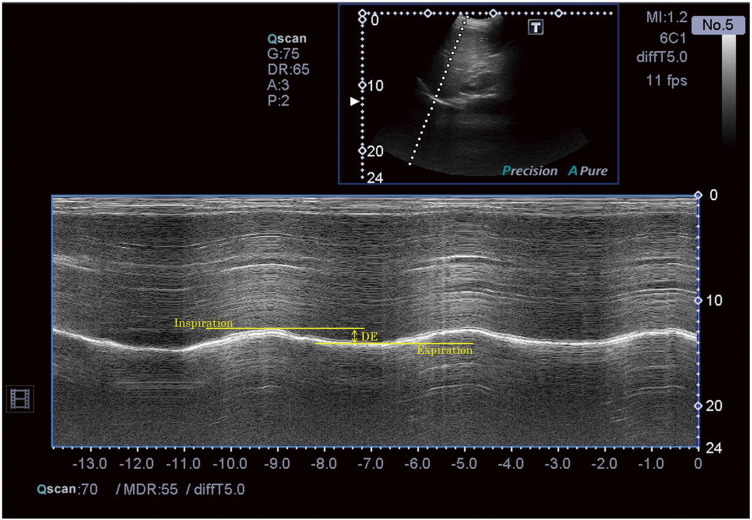
Measurement of DE DE, diaphragm excursion

DE was measured using ultrasound (Aplio 300; Canon Medhical Systems, Tochigi, Japan) and a 3.5 MHz convex probe, as described in a previous study^11)^. To ensure the reproducibility of the measurements, the kidneys were adjusted so that they could be displayed on the same screen in B mode^12)^. Measurements were conducted by a single, trained examiner to ensure reproducibility. The difference in distance from the body surface to the diaphragm observed during inspiration and expiration was measured in the M mode, and the difference between inspiration and expiration was defined as DE (Fig. 1). Measurements were taken once per person, and the VT and DE values were recorded over a 14-second interval. This duration was chosen based on the specifications of the ultrasound diagnostic equipment. During this period, every breath taken by the participant was recorded, ensuring that data from 2–4 respiratory cycles were captured and analyzed. This approach provided an accurate representation of diaphragmatic movement and tidal volume while minimizing short-term physiological variability. The 14-second interval was verified as sufficient for collecting reliable and reproducible data for all included breaths. All measurements were conducted in the seated position to ensure consistency and minimize postural effects on diaphragmatic motion and thoracic circumference.

The thoracic circumference of the lower thorax at maximal expiration was measured as the other index. It was measured on a horizontal plane passing through the intersection of the axillary midline and the tenth rib. The thoracic circumference at the level of the tenth rib is one of the indices most frequently used for measuring thoracic dilation during inspiration^13)^.

We estimated TVCs using 2 indices: TVC^a^ and TVC^b^. TVC^a^ was calculated as the product of DE and thoracic circumference, represented as TVC^a^ = DE × thoracic circumference. TVC^b^ was calculated as the product of DE and the square of the thoracic circumference, represented as TVC^b^ = DE × (thoracic circumference)^2^. The rationale for using these 2 indices lies in the foundation of basic geometric principles, which model TVC during resting respiration. TVC^a^ estimates the change in volume by considering the thoracic circumference as a linear measure of base and multiplying it by the DE, which represents the height. By contrast, TVC^b^ enhances this estimation by squaring the thoracic circumference to provide a more accurate approximation of the base area and then multiplying it by DE to achieve a closer representation of the cone volume mode.

When using only the chest circumference (TVC^a^), this calculation becomes a linear estimate of volume change and reflects the circumference of the base. However, when the chest circumference is squared (TVC^b^), a more accurate approximation of the cross-sectional area is obtained, and it is thought that this improves the prediction of volume change.

The normality of the data distribution was tested using the Shapiro–Wilk test before performing correlation analysis. Spearman’s rank correlation was employed due to non-normal distributions in some variables. Regression analyses were conducted with VT as the dependent variable, using DE, TVC^a^, and TVC^b^ as independent variables. Given the homogeneous age group, sex was treated as a potential covariate, and analyses were adjusted accordingly. Furthermore, regression analysis was performed with VT as the dependent variable, and prediction equations were developed using DE, TVC^a^, and TVC^b^ as covariates. SPSS ver27 (IBM Japan, Tokyo, Japan) was used for statistical analysis, with a statistical significance level of p <0.05.

## Results

Basic information about the participants’ physiques is shown in Table 1. The average thoracic circumference was 71.2 ± 4.7 cm.

**Table 1.** Characteristics of subjects

Age (years)	20.7 ± 1.0
Standing height (cm)	164.6 ± 8.2
Body weight (kg)	55.4 ± 5.4
BMI (kg/m^2^)	20.4 ± 1.6

Mean ± SD

BMI, body mass index; SD, standard deviation

The ventilatory parameters measured are shown in Table 2. The mean number of breaths measured in 14 seconds was 4.4 ± 1.7; the VT was 558.5 ± 146.8 mL; the DE was 13.1 ± 4.7 mm; the TVC^a^, expressed as DE × thoracic circumference (×10^3^), was 9.3 ± 3.4 mm^2^; and the TVC^b^, expressed as DE × thoracic circumference (×10^6^), was 6.7 ± 2.5 mm^3^.

**Table 2.** The ventilatory parameters

VT (mL)	558.5 ± 146.8
DE (mm)	13.1 ± 4.7
TCV^a^ (mm^2^) ×10^3^	9.3 ± 3.4
TCV^b^ (mm^3^) ×10^6^	6.7 ± 2.5

Mean ± SD

VT, tidal volume; DE, diaphragm excursion; TCV^a^, thoracic change volume (DE × thoracic circumference); TVC^b^, thoracic change volume (DE × thoracic circumference^2^); BMI, body mass index; SD, standard deviation

**Fig. 2.**
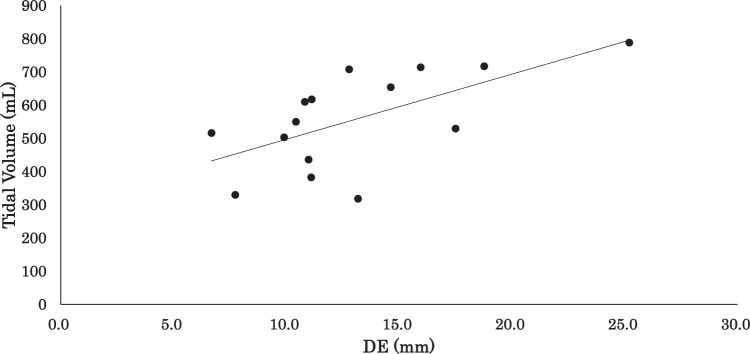
Relationship between tidal volume and DE DE, diaphragm excursion

**Fig. 3.**
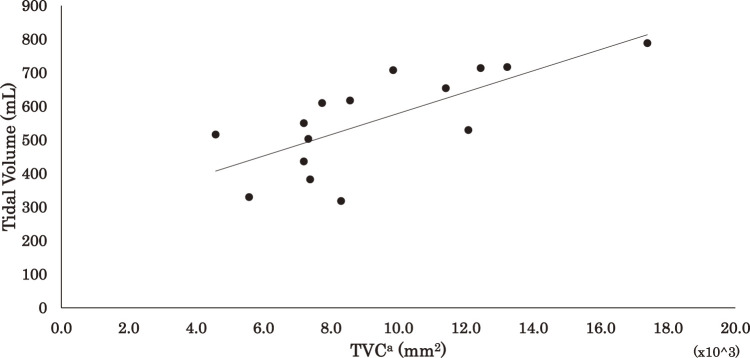
Relationship between tidal volume and TCV^a^ TCV^a^, thoracic change volume (diaphragm excursion × thoracic circumference)

**Fig. 4.**
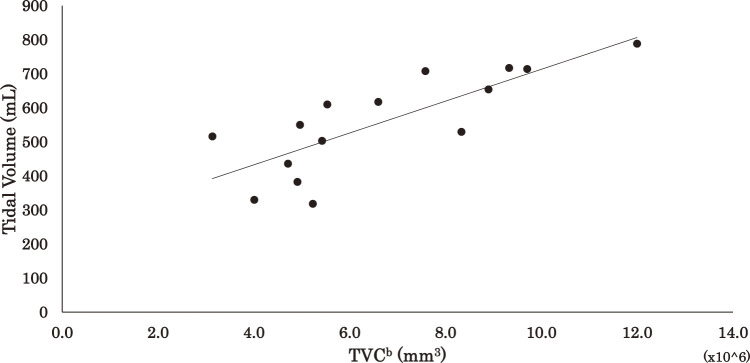
Relationship between tidal volume and TCV^b^ TVC^b^, thoracic change volume (diaphragm excursion × thoracic circumference^2^)

The results of the regression analysis are shown in Table 3 and Figs. 2–4 for the correlations between DE, TVC^a^, TVC^b^, and VT: ρ = 0.609 (p <0.05), ρ = 0.746 (p <0.01) and ρ = 0.839 (p <0.01), respectively.

**Table 3.** Tidal volume correlation with different variables

	ρ	p-value
DE	0.609	<0.01
TVC^a^	0.746	<0.01
TVC^b^	0.839	<0.01

DE, diaphragm excursion; TCV^a^, thoracic change volume (DE × thoracic circumference); TVC^b^, thoracic change volume (DE × thoracic circumference^2^)

The prediction equations and coefficients of determination for VT based on DE, TVC^a^, and TVC^b^ were as follows:

DE: VT = 19.6 * DE + 301.0 (R^2^ = 0.40)

TVC^a^ (DE × thoracic circumference): VT = 31.7 ×10^3^ × TVC^a^ + 262.1 (R^2^ = 0.53)

TVC^b^ (DE × thoracic circumference^2^): VT = 4.676 ×10^6^ × TVC^b^ + 245.7 (R^2^ = 0.63)

## Discussion

In this study, we estimated the TVC from the vertical diaphragm distance traveled and the thoracic circumference measured via ultrasound in healthy young adults and examined its relationship with VT. The basic inhalation mechanism involves the expansion of the thoracic cavity volume through 2 primary mechanisms: diaphragmatic contraction and rib elevation. The diaphragm, which is dome-shaped when relaxed, moves caudally during contraction, creating a volume change that can be approximated by a dome-shaped space. This volume is determined by the thoracic cavity cross-section at the diaphragm level (base) and the diaphragm movement distance (height). Our study focused primarily on diaphragmatic movement during resting breathing. However, it should be noted that the contribution of rib elevation to TVC may increase with greater ventilation volumes. For each relationship examined, a moderate correlation was found between DE and VT. By contrast, a stronger correlation was found between TVC and VT after taking into account the effect of body size. Body size was incorporated into the estimation model, using the square of the thoracic circumference to approximate the cross-sectional area of the thoracic cavity. This geometric adjustment offered a more precise representation of volume changes linked to DE, enhancing the predictive accuracy of VT.

The findings of this study highlight the advantages of employing a geometric approach over traditional methods relying solely on DE. By integrating thoracic circumference, the proposed model accounts for individual differences in body size, resulting in a more accurate prediction of VT. This improvement is particularly significant in clinical settings, where personalized assessments are critical. For example, the enhanced prediction capability of TVC^b^ underscores its potential to serve as a reliable, noninvasive tool for respiratory evaluation in diverse patient populations, including those with varying body compositions or limited ability to cooperate with traditional testing methods. The demonstrated predictive accuracy of TVC^b^ positions it as a novel and practical advancement in respiratory assessment methodologies.

The correlation between TVC^b^ (DE × thoracic circumference^2^) and VT (ρ = 0.839) was notably higher than that of DE alone (ρ = 0.609) or TVC^a^ (DE × thoracic circumference, ρ = 0.746). This underscores the improved predictive accuracy achieved by squaring the thoracic circumference, which aligns with the geometric principles underpinning the model. This geometric approach accounts for the nonlinear relationship between circumference and area, improving the prediction accuracy of VT. Cohen et al.^9)^ examined DE with variations in ventilation volume in a single individual, and the results of the present study, which examined multiple participants, may reflect differences in individual body size. Inhalation occurs as a result of a decrease in intrathoracic pressure due to expansion of the thoracic cavity volume caused by diaphragmatic contraction and rib elevation. The diaphragm is dome-shaped when relaxed and moves caudally when contracted. The TVC due to diaphragmatic movement is approximated by a dome-shaped volume with the thoracic cavity cross-section at the diaphragm elevation as the bottom and the diaphragm movement distance as the height. When the individual’s physique, especially the cross-sectional area of the bottom of the thoracic cavity, is different, the DE must be larger to ensure the same ventilation volume. This is because a smaller physique results in a smaller TVC. Therefore, it is considered that a higher correlation was obtained for TVC than for DE when people of different body sizes were included. It is geometrically obvious that the square of the thoracic circumference is the most appropriate alternative index for the cross-sectional area of the thoracic cavity.

The DE measurement used in this study is a standard ultrasound evaluation method^14–16)^, and thoracic circumference is typically measured at the tenth rib level. This methodology has not undergone significant changes in recent years. However, the combination of these measurements, as used in this study, has not been sufficiently validated, necessitating further verification in future research. Standardizing measurement protocols and refining calculation methods are crucial for improving the generalizability and reliability of this approach. Although this study used a single trained examiner to reduce variability, further research involving multiple examiners is necessary to validate reproducibility and ensure broader applicability.

The clinical applicability of the study results must be verified not only in healthy individuals but also in patients with respiratory diseases. It has been reported that DE decreases with increasing severity of respiratory disease, especially in patients with chronic obstructive pulmonary disease (COPD)^17)^. In addition, it is known that in patients with severe COPD, the diaphragm becomes flattened, and the efficiency of diaphragmatic respiration decreases, resulting in TVC, mainly due to rib motion^18)^. This measurement method is not capable of differentiating TVC due to diaphragmatic movement from that due to rib movement and may not reflect the ventilatory volume of COPD patients.

### Study limitations

Scarlata et al.^14)^ reported that DE during deep breathing was negatively correlated with age. Since the present study was limited to relatively young participants, we were not able to verify this point. However, the study by Scarlata et al.^14)^ did not control ventilation volume; therefore, further verification is needed to determine whether DE decreases even when the ventilation volume is equal.

This study specifically focused on healthy young adults to establish baseline correlations under controlled conditions. We recognize that the diaphragm and thoracic mechanics may differ significantly in individuals with obstructive or restrictive ventilatory disorders. Future research will extend these findings to clinical populations, but the present study’s design aimed to first establish validity in a homogeneous, healthy cohort.

Additionally, the TVC index used in this study failed to account for TVC associated with rib motion. It is necessary to further investigate the relationship when the ventilation volume is increased.

The results of this study may contribute to the development of a new method for assessing respiratory function using diaphragmatic movement. However, further validation is needed before this method can replace the spirometer which is currently used in clinical practice.

## Conclusions

The prediction of VT using ultrasound, which takes into account body size and TVC, had greater predictive ability than that using DE. In the future, the quantification of TVC associated with rib motion could further improve the prediction accuracy. Our study provides a new method for respiratory assessment, and future studies should explore integration with the quantification of rib motion, to further improve this promising method.

## Acknowledgments

We sincerely thank all the participants for their invaluable contributions to this study; their cooperation was essential to its completion. We are also deeply grateful to Prof. Shintarou Kudo for his expert guidance on ultrasound-based diaphragm imaging techniques, which significantly facilitated the execution of this research.

## Funding

Not applicable.

## Conflicts of Interest

The author declares that there are no conflicts of interest regarding the publication of this paper.

## References

[1] Anraku M Shargall Y: Surgical conditions of the diaphragm: anatomy and physiology. Thorac Surg Clin. 2009; 19: 419–429, v 20112625 10.1016/j.thorsurg.2009.08.002

[2] O’Donnell DE Revill SM, *et al.*: Dynamic hyperinflation and exercise intolerance in chronic obstructive pulmonary disease. Am J Respir Crit Care Med. 2001; 164: 770–777. 11549531 10.1164/ajrccm.164.5.2012122

[3] O’Donnell DE Voduc N, *et al.*: Effect of salmeterol on the ventilatory response to exercise in chronic obstructive pulmonary disease. Eur Respir J. 2004; 24: 86–94. 15293609 10.1183/09031936.04.00072703

[4] Graham BL Steenbruggen I, *et al.*: Standardization of spirometry 2019 update. An official American Thoracic Society and European Respiratory Society technical statement. Am J Respir Crit Care Med. 2019; 200: e70–e88. 31613151 10.1164/rccm.201908-1590ST PMC6794117

[5] Cardenas LZ Santana PV, *et al.*: Diaphragmatic ultrasound correlates with inspiratory muscle strength and pulmonary function in healthy subjects. Ultrasound Med Biol. 2018; 44: 786–793. 29373153 10.1016/j.ultrasmedbio.2017.11.020

[6] Grosu HB Ost DE, *et al.*: Diaphragm muscle thinning in subjects receiving mechanical ventilation and its effect on extubation. Respir Care. 2017; 62: 904–911. 28351903 10.4187/respcare.05370 PMC6373860

[7] DiNino E Gartman EJ, *et al.*: Diaphragm ultrasound as a predictor of successful extubation from mechanical ventilation. Thorax. 2014; 69: 423–427. 24365607 10.1136/thoraxjnl-2013-204111

[8] Liu M Jiang H, *et al.*: Tidal volume estimation using portable ultrasound imaging system. IEEE Sens J. 2016; 16: 9014–9020.

[9] Cohen E Mier A, *et al.*: Excursion-volume relation of the right hemidiaphragm measured by ultrasonography and respiratory airflow measurements. Thorax. 1994; 49: 885–889. 7940428 10.1136/thx.49.9.885 PMC475183

[10] Yamada Y Ueyama M, *et al.*: Time-resolved quantitative analysis of the diaphragms during tidal breathing in a standing position using dynamic chest radiography with a flat panel detector system (“Dynamic X-Ray Phrenicography”): initial experience in 172 volunteers. Acad Radiol. 2017; 24: 393–400. 27989446 10.1016/j.acra.2016.11.014

[11] Testa A Soldati G, *et al.*: Ultrasound M-mode assessment of diaphragmatic kinetics by anterior transverse scanning in healthy subjects. Ultrasound Med Biol. 2011; 37: 44–52. 21144957 10.1016/j.ultrasmedbio.2010.10.004

[12] Shiraishi M Higashimoto Y, *et al.*: Diaphragmatic excursion correlates with exercise capacity and dynamic hyperinflation in COPD patients. ERJ Open Res. 2020; 6: 00589–02020. 33447614 10.1183/23120541.00589-2020 PMC7792831

[13] Kaneko H Horie J: Breathing movements of the chest and abdominal wall in healthy subjects. Respir Care. 2012; 57: 1442–1451. 22348414 10.4187/respcare.01655

[14] Scarlata S Mancini D, *et al.*: Reproducibility and clinical correlates of supine diaphragmatic motion measured by M-mode ultrasonography in healthy volunteers. Respiration. 2018; 96: 259–266. 30114702 10.1159/000489229

[15] Boussuges A Gole Y, *et al.*: Diaphragmatic motion studied by M-mode ultrasonography: methods, reproducibility, and normal values. Chest. 2009; 135: 391–400. 19017880 10.1378/chest.08-1541

[16] Sferrazza Papa GF Pellegrino GM, *et al.*: A review of the ultrasound assessment of diaphragmatic function in clinical practice. Respiration. 2016; 91: 403–411. 27216909 10.1159/000446518

[17] Abd El Aziz AA Elwahsh RA, *et al.*: Diaphragmatic assessment in COPD patients by different modalities. Egypt J Chest Dis Tuberc. 2017; 66: 247–250.

[18] Gosselink RA Wagenaar RC, *et al.*: Diaphragmatic breathing reduces efficiency of breathing in patients with chronic obstructive pulmonary disease. Am J Respir Crit Care Med 1995; 151: 1136–1142. 7697243 10.1164/ajrccm.151.4.7697243

